# Successful control of vancomycin-resistant *Enterococcus faecium* outbreak in a neurosurgical unit at non-endemic region

**DOI:** 10.3134/ehtj.09.009

**Published:** 2010-03-31

**Authors:** V C C Cheng, J F W Chan, J W M Tai, Y Y Ho, I W S Li, K K W To, P L Ho, K Y Yuen

**Affiliations:** 1 Carol Yu Centre for Infection, Queen Mary Hospital, Hong Kong Special Administrative Region, China; 2 Infection Control Unit, Queen Mary Hospital, Hong Kong Special Administrative Region, China

## Abstract

Vancomycin-resistant enterococci (VRE) have emerged in many parts of the world, but have only been reported sporadically in Hong Kong. We report an outbreak of vancomycin-resistant *Enterococcus faecium* (VREfm) in a neurosurgical unit at a tertiary teaching hospital between 3 March and 3 April 2009 in Hong Kong. During the outbreak investigation, clinical samples from 193 (91.5%) of 211 patients who had stayed in the neurosurgical unit and 506 environmental samples were screened for VREfm. Besides the index case, another 3 (1.6%) out of 192 patients were found to be positive for VREfm. Two (0.4%) out of 506 environmental samples were positive for VREfm. All four clinical and two environmental isolates were found to be clonally related by pulse-field gel electrophoresis. The risk factors for nosocomial acquisition of VREfm included advanced age (*P*=0.047), presence of nasogastric tubing (*P*=0.002) and tracheostomy (*P*<0.001), and the use of β-lactam antibiotics (*P*<0.001) and vancomycin (*P*=0.001). Contrary to other VRE outbreaks in which the spread was rapid, the neurosurgical patients’ immobilization because of coma and mechanical ventilation dependency, and the vigilant practice of hand hygiene by health-care workers successfully limited the number of secondary cases despite the delayed recognition of the index case. All patients with VREfm were labeled in the hospital network information system so that stringent infection control measures with contact precautions would be carried out once these patients were readmitted to prevent its spread in our locality.

## Introduction

Since the initial reports of vancomycin-resistant enterococci (VRE) in France and the United Kingdom in 1986,^1^, ^2^ VRE have spread throughout the world and have become a major cause of nosocomial infections. During the 1990s, a significant increase of VRE was observed in the United States from 0.3% in 1989 to over 28% of all isolates in 2004.^3^, ^4^ Recently, VRE have emerged in Asian countries such as Singapore, Japan, Korea, and China 5–9 but have seldom been reported in Hong Kong. In an active surveillance study in a regional hospital between 2001 and 2002, only 1 out of 1792 patients being screened was found to harbor VRE in the stool.^10^ Reported risk factors for gastrointestinal colonization of VRE include hospitalization, residence in long-term care facilities, use of antibiotics, renal replacement therapy, and admission to high-risk clinical areas.^11^–^16^ Here, we report an outbreak of vancomycin-resistant *E. faecium* (VREfm) in our neurosurgical unit. Extensive investigation of the source, contact tracing of the potential secondary cases, and environmental surveillance were performed to control the spread of VREfm in a non-endemic area.

## Methods

### Epidemiological investigation

The neurosurgical unit at Queen Mary Hospital (hospital A), a university-affiliated teaching hospital in Hong Kong, serves as a tertiary referral center with the provision of acute neurosurgical and intensive care support in wards A7 and C7, respectively. Between 3 March and 3 April 2009, an outbreak of VREfm involving four cases occurred in wards A7 and C7 of hospital A. Contact tracing and screening culture of all patients who were discharged or transferred to convalescence hospitals (hospitals B, C, D, and E) during that period were performed. Environmental screening was done to assess the degree of contamination by sampling bedside rails, tables, lockers, bed linens, medical charts, drip stands, infusion pumps, blood pressure cuffs, bedpans, toilet facilities, and nursing stations (computer keyboards and controls, telephones, scanning machines, medical trolleys, and drug refrigerators) with premoistened swabs. Screening of stools for VREfm was offered to the relatives of patients with VREfm colonization. Cases were defined as those with positive culture of VREfm in either stool or rectal swabs. Their demographic data, clinical features, use of antibiotics, and co-morbidity were obtained using a standardized record form as previously described.^17^, ^18^ A case–control study was performed to assess the risk factors for acquisition of VREfm, with the control patients being selected as those who had been staying within the same cubicle as the cases in hospital A and B, but with a negative stool or rectal swab culture for VRE. The study was approved by the institutional review board.

### Laboratory investigation

Either stool or rectal swab with visible fecal component was collected, with stool being the preferred specimen. Cultures were performed within 24 h of specimen collection by inoculation onto chromogenic agar (chromID VRE, CHROMagar GRE). The Kirby–Bauer disk diffusion method and *E-test* (AB Biodisk, Solna, Sweden) were used to determine the susceptibility of the enterococci according to the Clinical and Laboratory Standards Institute or the manufacturer's instructions. Isolates with potential vancomycin resistance were confirmed by polymerase chain reaction as previously described.^19^ Primers for identification of *E. faecium* (F1 and F2 for *ddI*_*E*_. *faecium*) and the vancomycin resistance genotype (VanA, VanB, VanC1, and Van C23) were used.^20^ Pulsed-field gel electrophoresis (PFGE) of the clinical and environmental isolates was performed according to a standard protocol as previously described.^21^

### Statistical analysis

Chi-square test and Fisher's exact test were used in the analysis where appropriate. All reported *P*-values are two sided. *P*<0.05 was considered statistically significant in all analyses. Computations were performed with the use of SPSS version 15.0 for Windows.

## Results

### Epidemiological investigation

On 28 March 2009, VREfm was isolated from the catheterized urine in a 77-year-old man (patient 1) hospitalized in the neurosurgical unit (ward A7 and C7) of hospital A since 5 March 2009. The infection control team was informed and conducted an outbreak investigation. The first patient was immediately transferred into an isolation room with contact precautions and 28 other patients in the same unit were screened. VREfm were detected in the stool samples of two other patients including a 62-year-old woman (patient 2) and a 75-year-old man (patient 3). The starting date of the outbreak period was thus defined as 3 March 2009, the day on which patient 3 was admitted. Further contact tracing was performed which included 58 patients who had been transferred to the four convalescent hospitals since 3 March 2009 and remained hospitalized at the time of investigation. The VREfm was isolated from the stool of another 89-year-old man (patient 4) who was transferred to hospital B on 16 March 2009. Seventy-one out of 89 patients discharged from hospital A and another 35 patients staying with patient 4 in hospital B were traced and screened for VREfm. A total of 192 patients were screened with three (1.6%) of them being positive for VREfm. All patients confirmed to be VREfm positive were cared for in isolation rooms with contact precautions, and hand hygiene was enforced with emphasis on directly observed hand hygiene practice. Seven specimens from seven household members (one specimen each) of patients 1, 2, and 3 were found negative for VRE in voluntary screening. A total of 440 and 66 environmental samples were collected in hospital A and hospital B, respectively, and two of them taken in hospital B (bedside table and milk container) were positive for VREfm in both direct inoculation and after broth enrichment culture.

**Figure 1:**
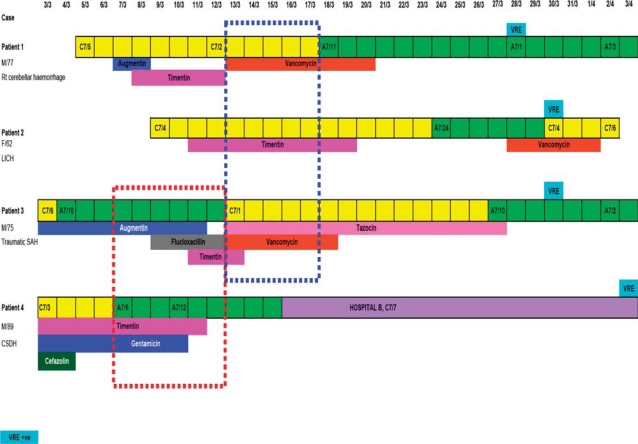
In-patient relationships and movements of the four patients with vancomycin-resistant *Enterococcus faecium* (VRE) in the neurosurgical unit of hospital A (unless specified) and details of their antimicrobial utilization. Solid blocks in yellow (C7) and green (A7)=days in which the patients stayed in the respective wards and beds in hospital A. Solid blocks in purple=days in which the patient stayed in hospital B. Dotted block in blue=days in which patients 1, 2, and 3 were staying in the same ward. Dotted block in red=days in which patients 3 and 4 were staying in the same ward.

Although patient 1 was the first identified case with both urine and gastrointestinal colonization of VREfm, epidemiological investigation showed that patient 4 could be the possible index case of this outbreak (Figure 1). He was directly transferred from a hospital in Mainland China and admitted to the neurosurgical intensive care unit in ward C7, bed number 3 (C7/3), for chronic subdural hematoma on 3 March 2009. He was given cefazolin as perioperative prophylaxis, and treated with intravenous ticarcillin–clavulanate and gentamicin for nosocomial pneumonia. When the index case was transferred to A7/6 and A7/12 on 7 March 2009 and 10 March 2009, respectively, the VREfm might have spread to patient 3 who was staying in A7/10 and receiving antimicrobial therapy for sepsis of unknown origin. When patient 3 deteriorated with clinical evidence of nosocomial pneumonia, he was transferred to C7/1 and was treated with intravenous piperacillin–tazobactam and vancomycin from 13 March 2009 onwards. The VREfm might have then further spread to patients 1 and 2 who were staying at C7/2 and C7/4, respectively. However, alternative sources and sequences of transmission were also possible as patients 1 and 4 had stayed in the same ward (C7/5 and C7/3) during the first few days of admission. Patient 4 was transferred to a convalescent hospital B on 16 March 2009 and detected to have VRE colonization in the stool on 2 April 2009. No secondary spread of VREfm was detected in the four convalescent hospitals receiving cases from the neurosurgical unit.

None of the four cases had symptoms suggestive of invasive VREfm infection. Except for patient 4 who died of his underlying disease, all patients recovered from the acute neurosurgical condition. These patients were labeled as being colonized by VREfm in the hospital network information system in order for the infection control team to implement appropriate measures if these patients are admitted to the hospital in future.

A case–control study was performed to identify the risk factors for nosocomial acquisition of VREfm (Table 1). There were four cases and 32 controls who were the patients staying in the same wards with the cases during their hospitalization in hospitals A and B. The statistically significant risk factors for acquisition included advanced age (*P*=0.047), presence of indwelling nasogastric tube (*P*=0.002) and endotracheal tube *(P*<0.001), and the use of antibiotics (*P*=0.001) during the contact period, particularly with β-lactams (*P*<0.001) and vancomycin (*P*=0.001). The difference in 30-day mortality after discharge was not statistically significant.

**Table 1:** Potential risk factors for acquisition of vancomycin-resistant *Enterococcus faecium* in the neurosurgical unit

	*Case (*n*=4)*	*Control (*n*=32)* ^a^	P*-value*
Age (mean)	75.8	55.8	0.047
Sex (male/female)	3:1	17:15	0.613
*Underlying disease*			
Cardiorespiratory disease	3	15	0.603
Malignancy	0	16	0.113
*Indwelling device*			
Nasogastric tubing	3	1	0.002
Tracheostomy	4	1	<0.001
Urinary catheter	3	13	0.303
*Current use of antimicrobials* ^*b*^			
All antimicrobials	4	2	0.001
β-lactam	4	2	<0.001
Vancomycin	3	0	0.001
Others	1	1	0.213

a 26 patients from hospital A and 6 patients from hospital B.

b At the time when the stool or rectal swab was collected.

### Laboratory investigation

**Figure 2:**
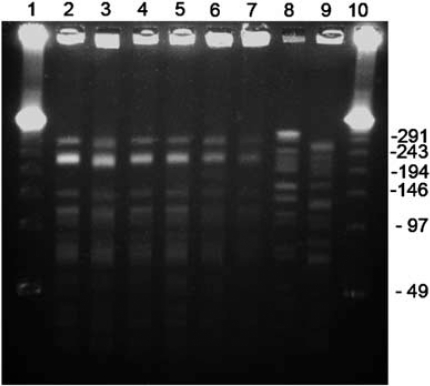
PFGE patterns of *Sma*I-digested DNAs of vancomycin-resistant *Enterococcus faecium*. Lane 1 and 10, molecular sizes are shown in kilobases; lane 2, patient 1 (8045), lane 3, patient 2 (8890); lane 4, patient 3 (8956); lane 5, patient 4 (1318); lane 6, environmental strain 1 (6318); lane 7, environmental strain 2 (6319); lane 8, unrelated patient strain 1 from another hospital (301036); lane 9, unrelated patient strain 2 from another hospital (304631).

The four isolates of *E. faecium* from patients’ stool or rectal swabs, and two isolates from the environment were characterized using a Vitek identification card. Their biochemical profiles and drug susceptibility patterns by disk diffusion test are listed in Table 2. All possessed *Van A* gene with minimum inhibitory concentration of higher than 256 mg/ml by *E-test*. PFGE analysis showed that all four clinical and two environmental isolates were clonally related (Figure 2).

**Table 2:** Laboratory characteristics of four isolates of vancomycin-resistant *Enterococcus faecium*

	*Patient 1*	*Patient 2*	*Patient 3*	*Patient 4*	*Environment 1*	*Environment 2*
Strain number	8045	8890	8956	1318	6318	6319
Date of collection	28 March 2009	30 March 2009	30 March 2009	3 April 2009	6 April 2009	6 April 2009
Specimen	Rectal swab	Rectal swab	Rectal swab	Stool	Bedside table	Milk container
Vitek identification	*E. faecium* (99%)	*E. faecium* (99%)	*E. faecium* (99%)	*E. faecium* (99%)	*E. faecium* (99%)	*E. faecium* (99%)
Vancomycin MIC (*E*-test)	>256 µg/ml	>256 µg/ml	>256 µg/ml	>256 µg/ml	*>*256 µg/ml	>256 µg/ml
Van A gene	Present	Present	Present	Present	Present	Present
Resistance pattern^a^	Amp, Gen,	Amp, Gen,	Amp, Gen,	Amp, Gen,	Amp, Gen,	Amp, Gen,
	Lev, Nit, Rif	Lev, Nit, Rif	Lev, Nit, Rif	Lev, Nit, Rif	Lev, Nit, Rif	Lev, Nit, Rif

a All the isolates were resistant to Amp, ampicillin; Gen, gentamicin (high content); Lev, levofloxacin; Nit, nitrofurantoin; Rif, rifampin; but susceptible to chlormaphenicol, fosfomycin, linezolid, minocycline, tetracycline, tigecycline, and streptomycin (high content).

## Discussion

VRE are uncommon in Hong Kong. Over the past 10 years, sporadically imported cases of VRE colonization or infection have been detected and outbreaks of limited scale have been reported in renal and orthopedic units (unpublished data). As enterococci constitute part of the normal gut flora, eradication after colonization in the gastrointestinal tract is very difficult and shedding of VRE can be for as long as 2 years.^22^ Therefore, whenever a case of colonization or infection is identified, stringent infection control measures are adopted to prevent their spread in our locality. However, it has become more difficult to maintain Hong Kong free of VRE as VRE have emerged in various parts of Asia. The increasing number of cases in Mainland China is of particular concern as there are occasional transferals of patients between the two areas.^8^, ^9^, ^23^ Our index case (patient 4), who had been hospitalized in Mainland China before returning to Hong Kong, might have acquired the infection there.

Prolonged utilization of broad-spectrum antibiotics may also predispose to VRE acquisition during hospitalization. The VRE colonization status of our index case was unknown because screening of stool or rectal swab for VRE was not routinely performed. The routine screening of VRE in nonendemic areas has not been found to be cost-effective because the microbial load of VRE may be well below the detection limit of standard culturing methods unless the patient is on antibiotics.^24^ The hypothesis that suppression of the gut flora by broad-spectrum antimicrobials would render patients susceptible to VRE colonization, is in accordance with our case–control analysis, which illustrates that the secondary colonized cases in the present outbreak may have acquired VRE while on antibiotics.^24^

The outbreak was identified when a catheterized urine specimen from patient 1 was positive for VRE. As patient 1 became bedridden after a neurosurgical operation and diaper care was required, contamination of the urinary catheter by fecal material may have occurred. To prevent the nosocomial transmission of VRE through the care of urinary catheter by health-care workers, catheterized urine was obtained from 25 hospitalized patients to ensure that this mode of transmission did not occur (data not shown). Three secondary cases were identified after contact tracing of almost 200 contacts, making the clinical attack rate less than 2%. This is much lower than the previously reported figure of more than 5%.^25^ There are a number of reasons to explain this apparent discrepancy. First, the immobilization of comatose and ventilator-dependent neurosurgical patients limited patient-driven environmental contamination. This was demonstrated by the limited number of positive cultures for VRE among our extensive environmental surveillance including more than 500 swabs. Only two environmental samples taken from a bedside table and a milk container were positive for the same strain of VREfm. The absence of diarrhea in all of our cases limited environmental contamination because the microbial load in the stool remained low during colonization.^26^ Enforcement of hand hygiene measures in our hospital also had a pivotal role in limiting the scale of both nosocomial transmission to patients and environmental contamination.^18^, ^27^, ^28^

The major limitation in this study is the small number of cases, which does not allow a more meaningful case–control analysis. On the other hand, the present data reinforces the importance of compliance to infection control measures and clinical alertness, especially when inter-hospital transferal of patients is involved, in preventing major outbreaks of VRE infection.^29^ Therefore, we advocate that stringent infection control measures of contact isolation and hand hygiene must be implemented even when colonization of VRE is apparently cleared. As shown in a previous study, VRE may become detectable when colonized patients are treated with antibiotics,^30^ which is similar to our past experiences involving multidrug-resistant *Staphylococcus* *aureus* (MRSA) and community-acquired MRSA where antibiotic treatment would unmask colonization status^.^ 31, 32
